# Invasion of the P elements: Tolerance is not futile

**DOI:** 10.1371/journal.pbio.3000036

**Published:** 2018-10-30

**Authors:** Colin D. Meiklejohn, Justin P. Blumenstiel

**Affiliations:** 1 School of Biological Sciences, University of Nebraska, Lincoln, Nebraska, United States of America; 2 Department of Ecology and Evolutionary Biology, University of Kansas, Lawrence, Kansas, United States of America

## Abstract

Organisms are locked in an eternal struggle with parasitic DNA sequences that live inside their genomes and wreak havoc on their host’s chromosomes as they spread through populations. To combat these parasites, host species have evolved elaborate mechanisms of resistance that suppress their activity. A new study in *Drosophila* indicates that, prior to the acquisition of resistance, individuals can vary in their ability to tolerate the activity of these genomic parasites, ignoring or repairing the damage they induce. This tolerance results from variation at genes involved in germline development and DNA damage checkpoints and suggests that these highly conserved cellular processes may be influenced by current and historical intragenomic parasite loads.

## Abstract 2

To combat parasitic DNA sequences, host species have evolved mechanisms of resistance that suppress their activity. This Primer discusses new evidence that, prior to the acquisition of resistance, individuals can vary in their ability to tolerate the activity of genomic parasites, ignoring or repairing the damage they induce.

In the early days of genetics, genomes were largely thought to be tranquil places. Genetic maps identified precise coordinates for genes that followed the rules of mendelian segregation. This view, however, did not last long. Beginning in the 1940s, Barbara McClintock's studies in maize revealed that the genome could be unruly [1,2]. Her groundbreaking work showed that autonomous and nonautonomous mobile elements could move throughout the genome, leaving behind them a trail of broken chromosomes and mutations. In the 1950s and ‘60s, studies using multiple species of *Drosophila* suggested that particular genotypes could have unstable genomes, with high rates of mutation and chromosomal breakage (for two excellent historical reviews, see [3,4]). In the 1960s, Mel Green showed that transposition also occurred in *Drosophila*, with genetically similar effects to those seen in maize [5]. By the 1970s, molecular genetic analysis in bacteria had begun to characterize mobile elements at the molecular level [6]. We now know that mobile genetic elements are ubiquitous and significant components of most eukaryotic genomes, comprising one-half of the nucleotides in our own chromosomes. The mutagenic and genotoxic effects of these nuclear parasites have driven the evolution of elaborate mechanisms of resistance in host eukaryotes that silence their activity. Now, a study published in *PLOS Biology* by Dr. Erin Kelleher and her colleagues shows how, rather than resisting, germlines can instead tolerate genomic damage wrought by mobile transposons [7].

## The mysterious P cytotype

In 1971, while crossing flies taken from natural populations with laboratory strains carrying visible markers, the *Drosophila* geneticist Yuichiro Hiraizumi observed unexpected recombination through males (crossing over is normally limited to the female germline in *Drosophila melanogaster*) [8]. Hirazumi and his colleagues showed that the factors causing male crossing over could be genetically mapped, and they designated these factors male recombination elements [9]. Shortly thereafter, Margaret Kidwell and John Sved independently discovered that male crossing over was a component of a broader phenomenon they termed hybrid dysgenesis [10]. Hybrid dysgenesis occurs when certain strains of *D*. *melanogaster* are crossed with one another, leading to a syndrome of sterility, mutation, chromosome breakage, male recombination, transmission distortion, and nondisjunction. Kidwell and Sved made the crucial discovery that hybrid dysgenesis was controlled by an interaction between the maternal cytoplasm and elements residing on the paternal chromosomes. This was revealed by the fact that when males of strains established from natural populations (P strains) were mated with laboratory strain females (M strains), hybrid dysgenesis would occur, while the reciprocal cross of P strain females with laboratory M strain males did not induce dysgenesis. This maternal effect was attributed to a hypothesized factor designated the "P cytotype."

In 1983, the molecular nature of P elements was revealed when white alleles obtained in dysgenic crosses were found to carry insertions of the same sequence [11]. Soon thereafter, the P element was harnessed as a tool for transgenesis, leading to a revolution in genetic engineering in an animal system. However, even as P elements were adopted and refined as a powerful tool for molecular genetics, the molecular basis for the P cytotype remained a mystery for two more decades. Genetic analysis showed that defective P elements encoding truncated transposases or missing promoter sequences could nonetheless confer a P cytotype and maintain repression of hybrid dysgenesis as long as these defective elements were present in the female germline [12,13]. Screens for P elements in natural populations found that independent P element insertions flanking the telomere of the X chromosome were potent suppressors of hybrid dysgenesis [11]. These telomeric P insertions were also found at high frequencies within natural populations [14], suggesting the possibility that natural selection was driving them to high frequency as a means to maintain P element control. But despite intense investigation, the molecular nature of the cytotype remained elusive, except for a putative "homology effect."

**Fig 1:**
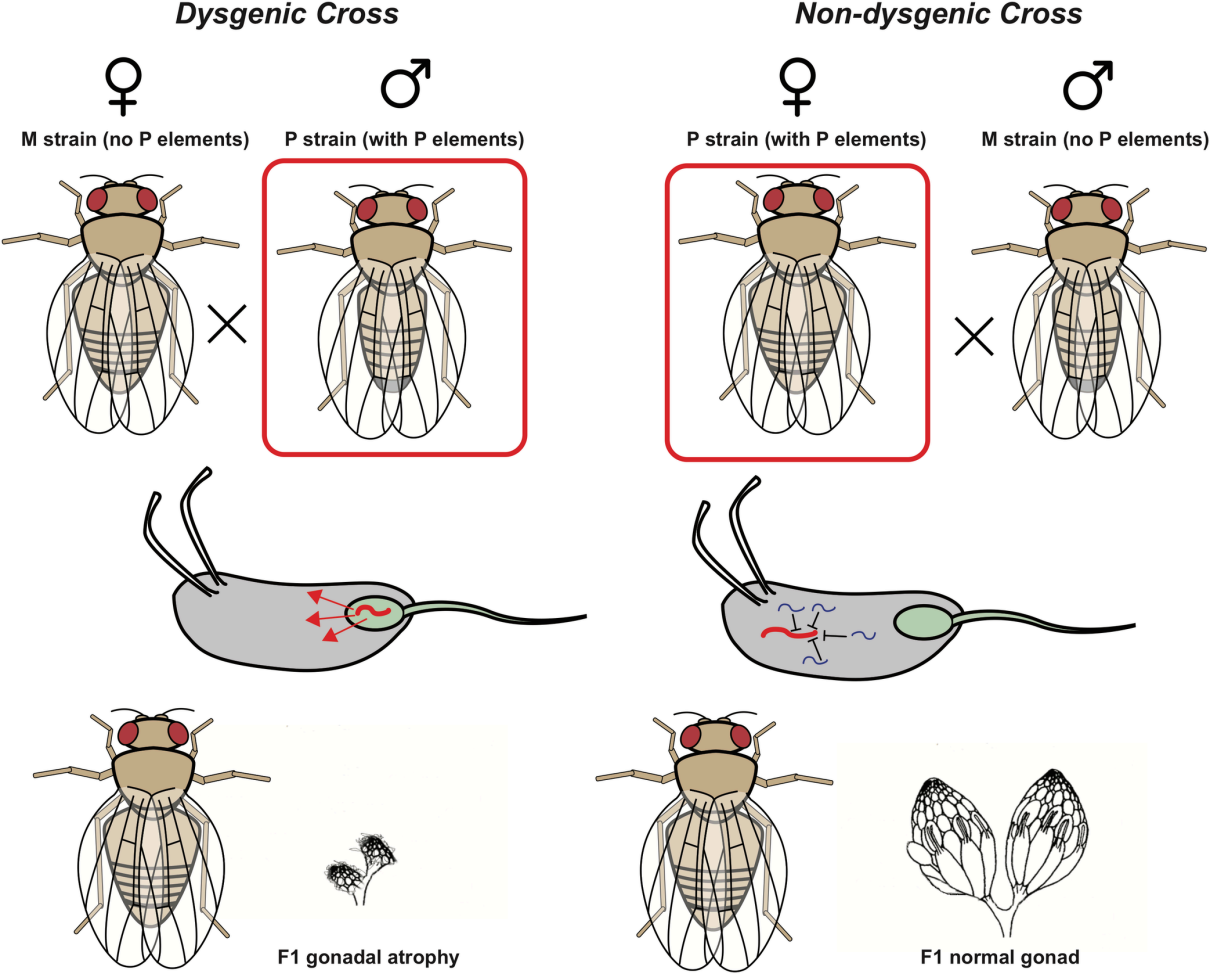
Maternally loaded piRNAs confer the P element cytotype and maintain P element resistance across generations. P–M dysgenesis is a syndrome of gonadal atrophy, male recombination, mutation, and chromosomal damage that occurs when P strain males are mated with M strain females. P strains carry P element transposons, and M strains do not. When P strain males fertilize the eggs of M strain females, P elements become mobilized in the germline. This mobilization causes early death of germline stem cells, and the offspring are sterile. In the reciprocal cross, when P strain females are mated with M strain males, repression of P elements is maintained, and progeny are fertile. In this cross, repression of P elements is maintained by the transmission of P element piRNAs in the female germline. In P strains, P element insertions in piRNA clusters provide a germline supply of P element piRNAs. This phenomenon constitutes a maternal effect because repression of dysgenesis can be maintained even if the progeny do not inherit the P strain alleles that are the source of piRNA. *Ovary image obtained from the public domain: https://en.wikipedia.org/wiki/File:DrosophilaOvary.png*. piRNA, piwi-interacting RNA.

The discovery of RNA interference in *Caenorhabditis elegans* and plants was the key to unlocking the molecular basis of the P cytotype. Soon thereafter, a complex pool of small RNAs derived from transposable element (TE) sequences was found to be present in the germline of *D*. *melanogaster* [15]. Curiously, these TE-derived small RNAs were slightly longer than the small interfering RNAs processed through the standard RNA silencing pathway. They were originally designated repeat-associated RNAs (rasiRNAs) until they were found to form complexes with Piwi proteins, giving them the new designation piwi-interacting RNAs (piRNAs). piRNAs are transcribed from discrete loci called piRNA clusters, where fragments of TE insertions generate a pool of antisense piRNA molecules that silence TEs and protect the genome [16]. In the case of the P cytotype, the P elements inserted near the telomere of the X chromosome are the source of piRNAs in the germline, and these piRNAs are transmitted maternally [17]. Moreover, maternal transmission of piRNAs is required to establish P element piRNA biogenesis in the next generation (Fig 1). Thus, maternal transmission of P element piRNAs maintained P element repression across generations. When P elements are inherited paternally, a maternal germline that lacks P element piRNAs is unable to maintain repression and the paternally transmitted P elements mobilize, causing germline DNA damage and the syndrome of hybrid dysgenesis. Laboratory stocks of *D*. *melanogaster* were established before the P element invaded wild populations of this species, hence the laboratory M cytotype.

Germline piRNAs that suppress TEs have now been found across metazoans [18]. The piRNA pathway is therefore an ancient genome defense mechanism conserved over hundreds of millions of years of evolution due to its ability to protect host genomes against the deleterious consequences of mobile genetic element activity. However, the mechanisms of piRNA biogenesis in *Drosophila* suggest that there may often be a significant lag between the invasion of a genome by a new transposable element and the establishment of suppressing piRNAs in the population. To produce the piRNAs that suppress the invader, the population must wait for the invading element to insert into a piRNA cluster locus and for these insertion alleles to reach high frequency. Some evolutionary models indicate that selection for alleles that suppress TE movement is weak [19], which could further delay the spread of resistance. Therefore, following the invasion of a new TE but prior to the acquisition of piRNA-mediated resistance, the fitness of host individuals may be determined by their ability to tolerate transposable element activity in their genome. Until recently, the existence and nature of tolerance to TE activity has been overlooked. Now, a new study published in *PLOS Biology* by Dr. Erin Kelleher and her colleagues has combined the P–M hybrid dysgenesis system with a powerful quantitative genetic resource to study the genetic basis for tolerance to P element transposition in *D*. *melanogaster*. This study provides new insight into how the germline can be robust to ongoing genomic damage in the presence of an invading transposon.

## Tolerant genomes

Kelleher and colleagues utilized the Drosophila Synthetic Population Resource (DSPR) [20]—a set of fully genotyped recombinant inbred lines generated from eight founder strains—to characterize and map genetic variation affecting female tolerance to P element transposon activity. The eight DSPR founder strains were brought into research labs prior to the P element invasion of natural *D*. *melanogaster* populations so are free of P elements and are susceptible to P element–induced hybrid dysgenesis. Kelleher and colleagues crossed DSPR females to males from a strain that is a strong inducer of hybrid dysgenesis and examined F_1_ females for the presence of normal or atrophied ovaries, the latter phenotype indicative of hybrid dysgenesis. At high temperatures, such dysgenic crosses produce uniform and complete ovarian atrophy, so these crosses were done at a lower, permissive temperature to reveal variation in the degree of dysgenesis. Differences in the degree of hybrid dysgenesis between F_1_ females from different DSPR strains indicated the existence of genetic variation affecting tolerance to P element mobilization.

**Fig 2:**
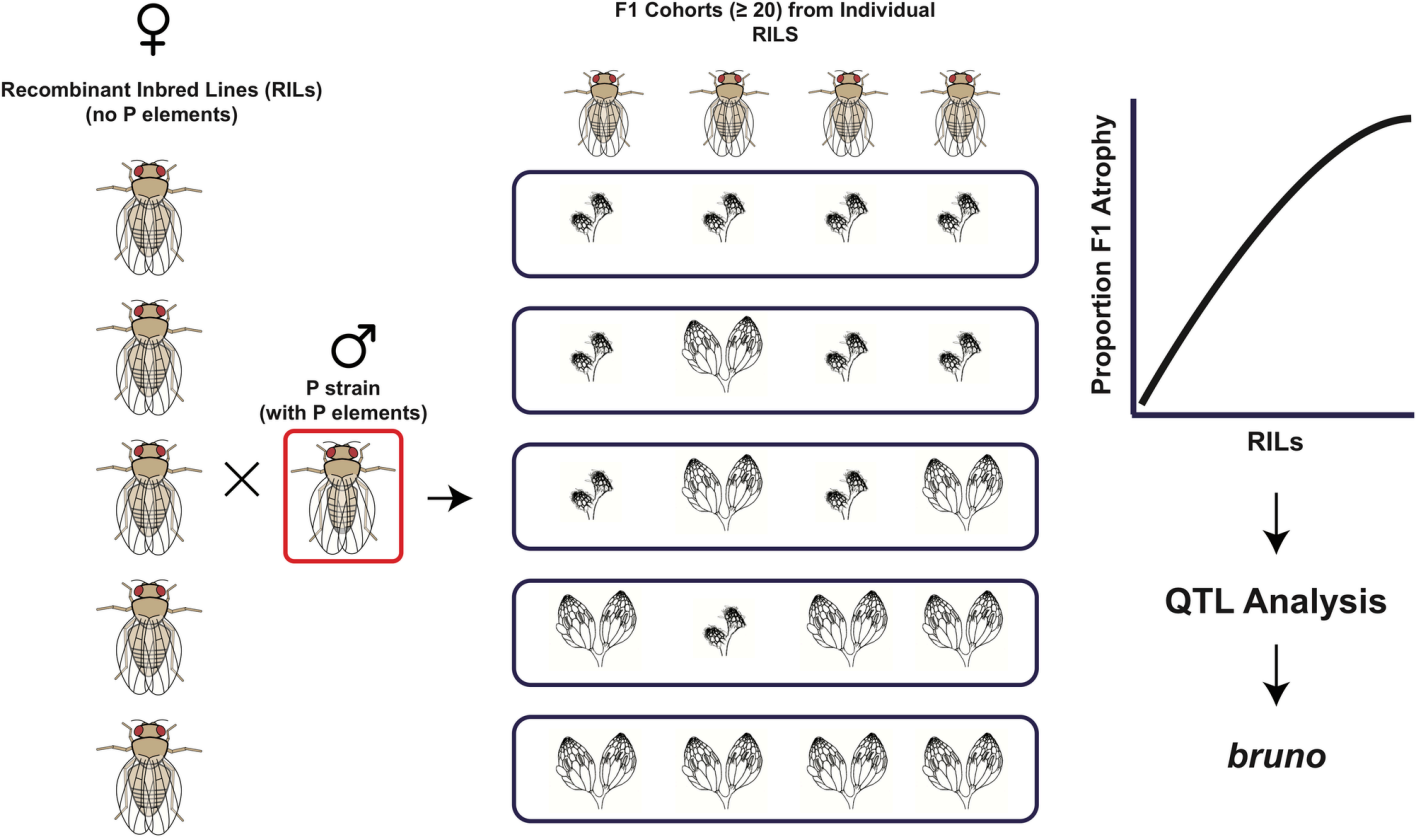
QTL analysis reveals variation in *bruno* mediates natural tolerance to P element–induced gonadal atrophy. Natural variation in resistance to hybrid dysgenesis was identified using a large number of RILs from the DSPR. A key feature of the DSPR is that it was constructed from strains that lack P element insertions and, hence, also lack P element piRNAs. Multiple females from each RIL were crossed to males of a P strain with a strong capacity to induce dysgenesis. Variation in tolerance to P element–induced atrophy was revealed by performing crosses at a mildly permissive temperature. This experiment revealed a tremendous amount of variation: some strains exhibit no gonadal atrophy, while others exhibit complete gonadal atrophy. Using this variation in a QTL analysis, segregating variation at the *bruno* gene was found to be an important determinant of tolerance to P element–induced sterility in the DSPR. DSPR, Drosophila Synthetic Population Resource; QTL, quantitative trait locus; RIL, recombinant inbred line.

To phenotype replicate individuals from 660 DSPR genotypes, Kelleher and colleagues scored the proportion of replicate females of each genotype that had at least one mature egg chamber. This phenotype showed the full range of variation: in some genotypes, 100% of females assayed had zero mature egg chambers, while in other genotypes, all females had at least one mature egg chamber (Fig 2). Kelleher and colleagues estimated the broad-sense heritability of tolerance in this mapping population to be 40%, suggesting that such genetic variation is likely segregating in natural populations and could respond to selection following the invasion of a new transposable element. Furthermore, there is likely to be additional genetic variation associated with quantitative differences in fecundity between genotypes that was not assayed in this study. Standing genetic variation and selection for tolerance to transposable element activity may therefore be an important component of population responses to these parasites.

The fully genotyped DSPR strains were designed for quantitative trait locus mapping, and using this approach, Kelleher and colleagues discovered a 300-kb region on Chromosome 2L that strongly influences the proportion of ovarian atrophy across DSPR genotypes. Within this region, they identified just one gene that is both highly expressed in the ovary and contains polymorphisms that segregate in phase with phenotypic variation (i.e., polymorphisms that differentiate resistant and sensitive haplotypes). This gene, *bruno*, encodes an RNA-binding protein that is required for both germline differentiation in the ovary [21] and proper patterning of the embryo [22]. Two additional pieces of evidence implicate *bruno* as a tolerance locus in the DSPR. First, gene expression assays indicate that tolerant alleles of *bruno* have approximately 20% lower transcript levels than intolerant alleles in six of the DSPR lines. Second, loss-of-function and deletion alleles of *bruno* greatly increase tolerance to P element dysgenesis. Together, these data suggest that *bruno* function is inversely correlated with P element tolerance and that regulatory variation at this gene leads to variation in tolerance to dysgenesis in the DSPR mapping population.

## Beyond *bruno*

The identification and characterization of genetic variation for tolerance to TE activity opens the door for studies into the mechanisms and evolution of this host–parasite interaction. One question immediately raised by the results of this study is how variation in *bruno* function leads to variation in tolerance. One hypothesis put forward by Kelleher and colleagues is that the balance between germline stem cell renewal and differentiation in the ovary is disrupted by TE-induced DNA damage, leading to the loss of stem cells and ovarian atrophy. *bruno* promotes stem cell differentiation, so reduced *bruno* activity could lead to increased retention of stem cells in the niche, ultimately generating tolerance to DNA damage. This hypothesis is supported by experiments showing that mutations in genes required for the DNA damage response also modify the degree of hybrid dysgenesis [23]. This in turn raises the possibilities that both the optimal stringency of DNA damage checkpoints and regulation of germline stem cells could be influenced by current levels of TE activity within a population and that variation in TE tolerance could contribute to natural variation in fertility.

P–M hybrid dysgenesis has been a powerful system for studying the interplay between TEs and their hosts, but it is not clear how representative this system is of such interactions in nature. This is because the dysgenesis syndrome results from an extreme difference in TE burden caused by the sequestration of *D*. *melanogaster* strains in research laboratories, while the ancestral population outside was infected. Population structure can influence rates of TE spread through a species, but very low migration rates would be required to keep a subpopulation completely free of transposable elements that are infecting neighboring demes [24]. More often, matings will occur between individuals with modest differences in the number of active TEs they carry. It is unknown whether quantitative differences in TE burden can cause milder derepression of TE activity and concomitant reductions in fertility than seen in P–M dysgenesis. In the laboratory, it is difficult to detect the fertility effects of minor differences in germline stem cell number that might result from such quantitative differences in TE burden. However, in large populations, such differences will be visible to natural selection and are expected to shape the "decision" a stem cell must make to repair its DNA or undergo cell death when transposition occurs outside of the context of hybrid dysgenesis. Thus, although Kelleher and colleagues’ study used P–M dysgenesis as a sensitized system, the genetic variation it uncovered may nonetheless be an important target for selection in the early stages of invasion or in later stages when transposition rates are low.

More generally, both theory and data suggest that tolerance to parasites may be a widespread and evolutionarily significant strategy for host organisms. Resistance to parasites decreases infection frequency within populations, and this negative feedback can impede the spread of resistance traits. In contrast, tolerance to parasites increases infection frequency and promotes the spread and fixation of tolerance traits [25] and typically does not select for coevolutionary responses from pathogens. The significance of tolerance is exemplified by the attenuated immune response of green monkeys to simian immunodeficiency virus, which allows green monkey populations to experience high infection prevalence and viremia with no loss of health [26]. Tolerance to parasites may therefore be a more successful long-term strategy than resistance, and by allowing host populations to coexist with their parasites, tolerance could potentially facilitate transitions between parasitic and mutualistic interactions. Eukaryotic genomes show signatures of transposable element families that were active for millions of years [27], perhaps as a result of host tolerance to these mobile genetic elements' activity. Such patterns raise the question of whether TE invasions occur frequently enough to produce signatures of recurrent selection at genes like *bruno*, with the relative fitness of more and less tolerant alleles determined by fluctuating burdens of active transposable elements. The P element alone has colonized the genomes of at least two species of *Drosophila* within the last 100 years [28,29], indicating that such invasions are not rare. How these dynamics of resistance and tolerance might differ between infectious, horizontally transmitted parasites and vertically transmitted genetic parasites is unclear. Investigating these and related questions will illuminate not only the nature of interactions between these ubiquitous genetic parasites and their hosts but may more broadly inform our understanding of parasite burden, health, and disease.

## Abbreviations

DSPR: Drosophila Synthetic Population Resource
piRNA: piwi-interacting RNA
rasiRNA: repeat-associated RNA
TE: transposable element

## References

[1] McClintock B. Mutable loci in maize. Year B Carnegie Inst Wash. 1948;47: 155–169.

[2] McClintock B. The origin and behavior of mutable loci in maize. Proc Natl Acad Sci U S A. 1950;36: 344–355. 15430309 10.1073/pnas.36.6.344 PMC1063197

[3] Crow JF. The genesis of dysgenesis. Genetics. 1988;120: 315–318. 17246479 10.1093/genetics/120.2.315 PMC1203511

[4] Engels WR. Invasions of P elements. Genetics. 1997;145: 11–15. 9017385 10.1093/genetics/145.1.11 PMC1207769

[5] Green MM. Controlling element mediated transpositions of the white gene in Drosophila melanogaster. Genetics. 1969;61: 429–441. 5807807 10.1093/genetics/61.2.429 PMC1212168

[6] Galas DJ, Chandler M. Bacterial Insertion Sequences. Mob DNA. American Society of Microbiology; 1989; 109–192.

[7] Kelleher ES, Jaweria J, Akoma U, Ortega L, Tang W. QTL mapping of natural variation reveals that the developmental regulator *bruno* reduces tolerance to P-element transposition in the *Drosophila* female germline. PLoS Biol. 2018;16(10):e2006040 10.1371/journal.pbio.2006040 PMC6207299 30376574

[8] Hiraizumi Y. Spontaneous recombination in Drosophila melanogaster males. Proc Natl Acad Sci U S A. 1971;68: 268–270. 5277066 10.1073/pnas.68.2.268 PMC388914

[9] Slatko BE, Hiraizumi Y. Elements causing male crossing over in Drosophila melanogaster. Genetics. 1975;81: 313–324. 812770 10.1093/genetics/81.2.313 PMC1213400

[10] Kidwell MG, Kidwell JF, Sved JA. Hybrid Dysgenesis in Drosophila melanogaster: A Syndrome of Aberrant Traits Including Mutation, Sterility and Male Recombination. Genetics. 1977;86: 813–833. 17248751 10.1093/genetics/86.4.813 PMC1213713

[11] Rubin GM, Kidwell MG, Bingham PM. The molecular basis of P-M hybrid dysgenesis: the nature of induced mutations. Cell. 1982;29: 987–994. 6295640 10.1016/0092-8674(82)90462-7

[12] Ronsseray S, Josse T, Boivin A, Anxolabéhère D. Telomeric transgenes and trans-silencing in Drosophila. Genetica. 2003;117: 327–335. 12723712 10.1023/a:1022929121828

[13] Niemi JB, Raymond JD, Patrek R, Simmons MJ. Establishment and maintenance of the P cytotype associated with telomeric P elements in Drosophila melanogaster. Genetics. 2004;166: 255–264. 15020423 10.1534/genetics.166.1.255 PMC1470675

[14] Ajioka JW, Eanes WF. The accumulation of P-elements on the tip of the X chromosome in populations of Drosophila melanogaster. Genet Res. Cambridge University Press; 1989;53: 1–6. 2497050 10.1017/s0016672300027798

[15] Aravin AA, Lagos-Quintana M, Yalcin A, Zavolan M, Marks D, Snyder B, et al The small RNA profile during Drosophila melanogaster development. Dev Cell. 2003;5: 337–350. 12919683 10.1016/s1534-5807(03)00228-4

[16] Brennecke J, Aravin AA, Stark A, Dus M, Kellis M, Sachidanandam R, et al Discrete small RNA-generating loci as master regulators of transposon activity in Drosophila. Cell. 2007;128: 1089–1103. 10.1016/j.cell.2007.01.043 17346786

[17] Brennecke J, Malone CD, Aravin AA, Sachidanandam R, Stark A, Hannon GJ. An epigenetic role for maternally inherited piRNAs in transposon silencing. Science. 2008;322: 1387–1392. 10.1126/science.1165171 19039138 PMC2805124

[18] Saito K, Siomi MC. Small RNA-mediated quiescence of transposable elements in animals. Dev Cell. 2010;19: 687–697. 10.1016/j.devcel.2010.10.011 21074719

[19] Nuzhdin SV. Sure facts, speculations, and open questions about the evolution of transposable element copy number. Genetica. 1999;107: 129–137. 10952206

[20] King EG, Merkes CM, McNeil CL, Hoofer SR, Sen S, Broman KW, et al Genetic dissection of a model complex trait using the Drosophila Synthetic Population Resource. Genome Res. 2012;22: 1558–1566. 10.1101/gr.134031.111 22496517 PMC3409269

[21] Schüpbach T, Wieschaus E. Female sterile mutations on the second chromosome of Drosophila melanogaster. II. Mutations blocking oogenesis or altering egg morphology. Genetics. 1991;129: 1119–1136. 1783295 10.1093/genetics/129.4.1119 PMC1204776

[22] Kim-Ha J, Kerr K, Macdonald PM. Translational regulation of oskar mRNA by bruno, an ovarian RNA-binding protein, is essential. Cell. 1995;81: 403–412. 7736592 10.1016/0092-8674(95)90393-3

[23] Tasnim S, Kelleher ES. p53 is required for female germline stem cell maintenance in P-element hybrid dysgenesis. Dev Biol. 2018;434: 215–220. 10.1016/j.ydbio.2017.12.021 29294306

[24] Deceliere G, Charles S, Biémont C. The dynamics of transposable elements in structured populations. Genetics. 2005;169: 467–474. 10.1534/genetics.104.032243 15466430 PMC1448865

[25] Roy BA, Kirchner JW. Evolutionary dynamics of pathogen resistance and tolerance. Evolution. 2000;54: 51–63. 10937183 10.1111/j.0014-3820.2000.tb00007.x

[26] Chahroudi A, Bosinger SE, Vanderford TH, Paiardini M, Silvestri G. Natural SIV hosts: showing AIDS the door. Science. 2012;335: 1188–1193. 10.1126/science.1217550 22403383 PMC3822437

[27] Pace JK 2nd, Feschotte C. The evolutionary history of human DNA transposons: evidence for intense activity in the primate lineage. Genome Res. 2007;17: 422–432. 10.1101/gr.5826307 17339369 PMC1832089

[28] Daniels SB, Peterson KR, Strausbaugh LD, Kidwell MG, Chovnick A. Evidence for horizontal transmission of the P transposable element between Drosophila species. Genetics. 1990;124: 339–355. 2155157 10.1093/genetics/124.2.339 PMC1203926

[29] Kofler R, Hill T, Nolte V, Betancourt AJ, Schlötterer C. The recent invasion of natural Drosophila simulans populations by the P-element. Proc Natl Acad Sci U S A. 2015;112: 6659–6663. 10.1073/pnas.1500758112 25964349 PMC4450375

